# Molecular and SEM studies on *Thaparocleidus vistulensis* (Siwak, 1932) (Monopisthocotyla, Ancylodiscoididae)

**DOI:** 10.1038/s41598-024-61032-3

**Published:** 2024-05-04

**Authors:** Wan Muhammad Hazim Wan Sajiri, Csaba Székely, Kálmán Molnár, Sebastian Kjeldgaard-Nintemann, Per Walter Kania, Kurt Buchmann, Boglárka Sellyei

**Affiliations:** 1 HUN-REN Veterinary Medical Research Institute, 21, Hungária Krt, 1143 Budapest, Hungary; 2 Doctoral School of Animal Biotechnology and Animal Science (Agricultural Science), Hungarian University of Agriculture and Life Sciences, 1. Páter Károly Str, 2100 Gödöllő, Hungary; 3 Center for Advanced Bioimaging, University of Copenhagen, Frederiksberg C, Denmark; 4 Department of Veterinary and Animal Sciences, Faculty of Health and Medical Sciences, University of Copenhagen, Frederiksberg C, Denmark

**Keywords:** *Thaparocleidus vistulensis*, *Silurus glanis*, Sclerotized structure, Male copulatory organ, Molecular analysis, rDNA, Molecular biology, Structural biology, Zoology

## Abstract

Presenting new molecular and scanning electron microscope (SEM) features, this study gives additional data to the better knowledge of *Thaparocleidus vistulensis* (Siwak, 1932) (Monopisthocotyla, Ancylodiscoididae), a parasite of the European catfish *Silurus glanis* Linnaeus, 1758 (Siluriformes, Siluridae) cultured in a commercial fish farm in Hungary. In addition, notes on the early development of sclerotized anchors are also provided. The main morphological difference of *T. vistulensis* compared to other congeneric species is associated with the male copulatory organ, which exhibits 5–7 loops in the middle of the penis length and a long open V-shaped sclerotized accessory piece, dividing terminally into two parts, securing the terminal part of the penis tube. The present study provides for the first time molecular characterization data based on the 2694 bp long nucleotide sequence of rDNA (ITS1, 5.8S, ITS2, and flanked with partial 18S and partial 28S) submitted in GenBank with the accession number OR916383. A phylogenetic tree based on ITS1 sequences supports a well-defined clade including *T. vistulensis*, forming a sister group with *T. siluri,* a species-specific monopisthocotylan parasite to *S. glanis*. The morphological characterization of *T. vistulensis,* especially for the male copulatory organ, together with the molecular data in the present study, extends knowledge about this monopisthocotylan species and provides new information for future phylogeny studies.

## Introduction

Monogeneans are considered the most diverse parasite group with regard to the number of species, morphology, and ecology^1,2^. These parasites exhibit a high host specificity, parasitizing a single or a narrow group of closely related fishes^2^. Monogenean parasites are equipped with a distinctive posterior attachment structure, the opisthaptor, utilized for anchoring to the host surface (body, fins, and gills). This conspicuous organ is equipped with various types of sclerotized hooks and anchors^3^, which is, together with sclerotized structures in the male copulatory organ and the vagina, decisive for species differentiation^4^.

Monogenea is considered as a non-monophyletic group based on previous phylogenetic studies including mtDNA^5^ and rDNA^6^. Transcriptomic data studied by Brabec et al.^7^ showed robust and consistent signals in the two non-monophyly monogenean lineages ﻿(subclasses: Monopisthocotylea and Polyopisthocotylea). Despite the term use of conventionally recognized as class “Monogenea” being common in the nomenclature of phylum Platyhelminthes, Brabec et al.^7^ proposed to suppress the term and promote the previously subclasses to the class level as Monopisthocotyla new class and Polyopisthocotyla new class. Hence, this terminology will be used here.

*Thaparocleidus vistulensis* (Siwak, 1932) is a monopisthocotylan ectoparasite occurring on the gills of *Silurus glanis* Linnaeus, 1758. Firstly, it was described by Siwak^8^ from Poland as *Ancyrocephalus vistulensis*. Roman^9^ recorded the species synonym with *Ancylodiscoides siluri*, a synonymization later repeated by Roman-Chiriac^10^. However, it was rejected by Yamaguti^11^. Several changes of genera occurred until Lim^12^ reassessed and transferred them to *Thaparocleidus*. The species was reported infecting the same host (*Silurus glanis*) from Hungary^13,14^, Czechia^15,16^, Iraq^17^, Iran^18,19^, Turkey^20^, Italy^21,22^, Poland^23^, and UK^24^. In the last century, several descriptions were reported, mainly based on morphological characteristics^22,25^. Nevertheless, it deserves more attention due to the complicated structure of its male and female copulatory organs.

Although morphological studies form the basis of correct species identification, molecular data is still vital in supporting a complete and conclusive identification^26^. To date, the description of *T. vistulensis* is based on morphological details and does not provide a molecular characterization of the species described except in^27^, and the sequence information available at the International Nucleotide Sequence Database Collaboration (INSDC) is limited. This study re-evaluates morphometric data with a specific focus on the haptoral and copulatory sclerites, and presents new molecular data and SEM studies, extending current knowledge on the species.

## Results

### Morphological description

**Figure 1:**
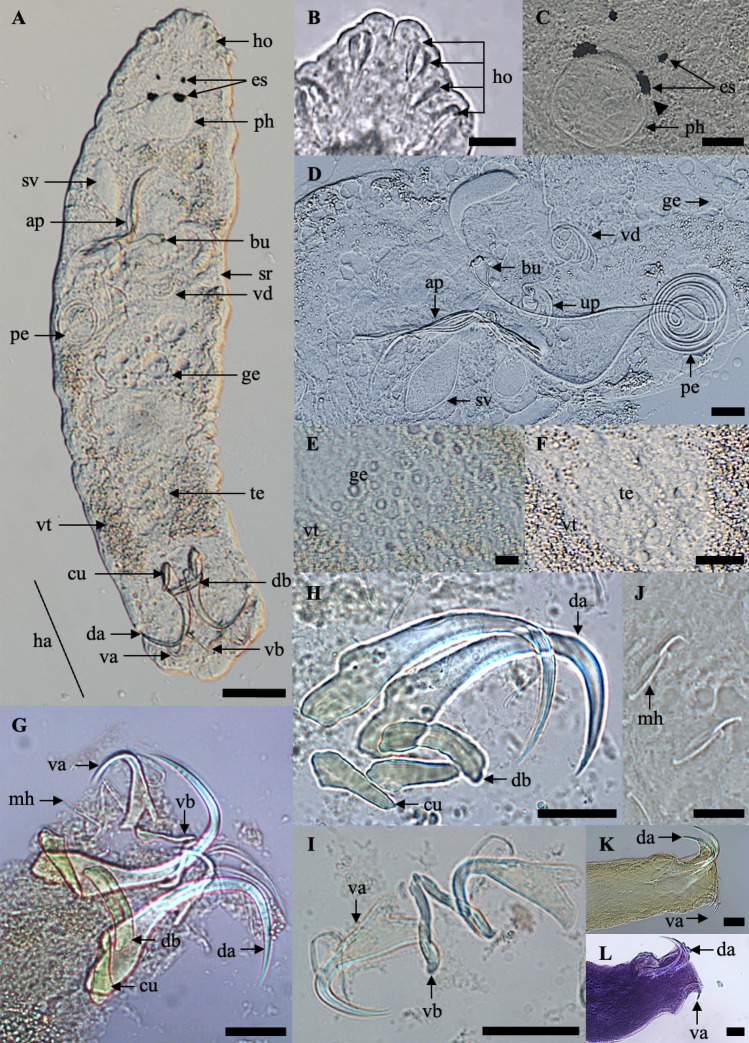
Photomicrograph of *Thaparocleidus vistulensis*. (**A**) Whole mount—dorsal view; (**B**–**C**) Anterior region; (**D**) Complex internal organ; (**E**) Germarium; (**F**) Testis; (**G**) Opisthaptor; (**H**) Dorsal anchor with cuneus and dorsal bar; (**I**) Marginal hooks; (**J**) Ventral anchor and ventral bar; (**K**–**L**) Haptor—lateral view. (**A**–**F**) Fresh samples; (**G**–**J**) Softened with proteinase K and mounted in glycerine-ammonium-picrate; (K) Mounted in glycerine-ammonium-picrate; (**L**) Stained with hematoxylin. Abbreviations: ap, accessory piece; bu, bulbous base; cu, cuneus; da, dorsal anchor; db, dorsal bar; es, eye spots; ge, germarium; ha, haptor; ho, head organs; mh, marginal hooks; pe, penis; ph, pharynx; sr, seminal receptacle; sv, seminal vesicle; te, testis; up, uterine pore; va, ventral anchor; vb, ventral bar; vd, vaginal duct; vt, vitellaria. ▲, transparent and intensely refractive corpuscles. Scale bars represent 20 μm, except (A) 100 μm and (J) 10 μm.

**Figure 2:**
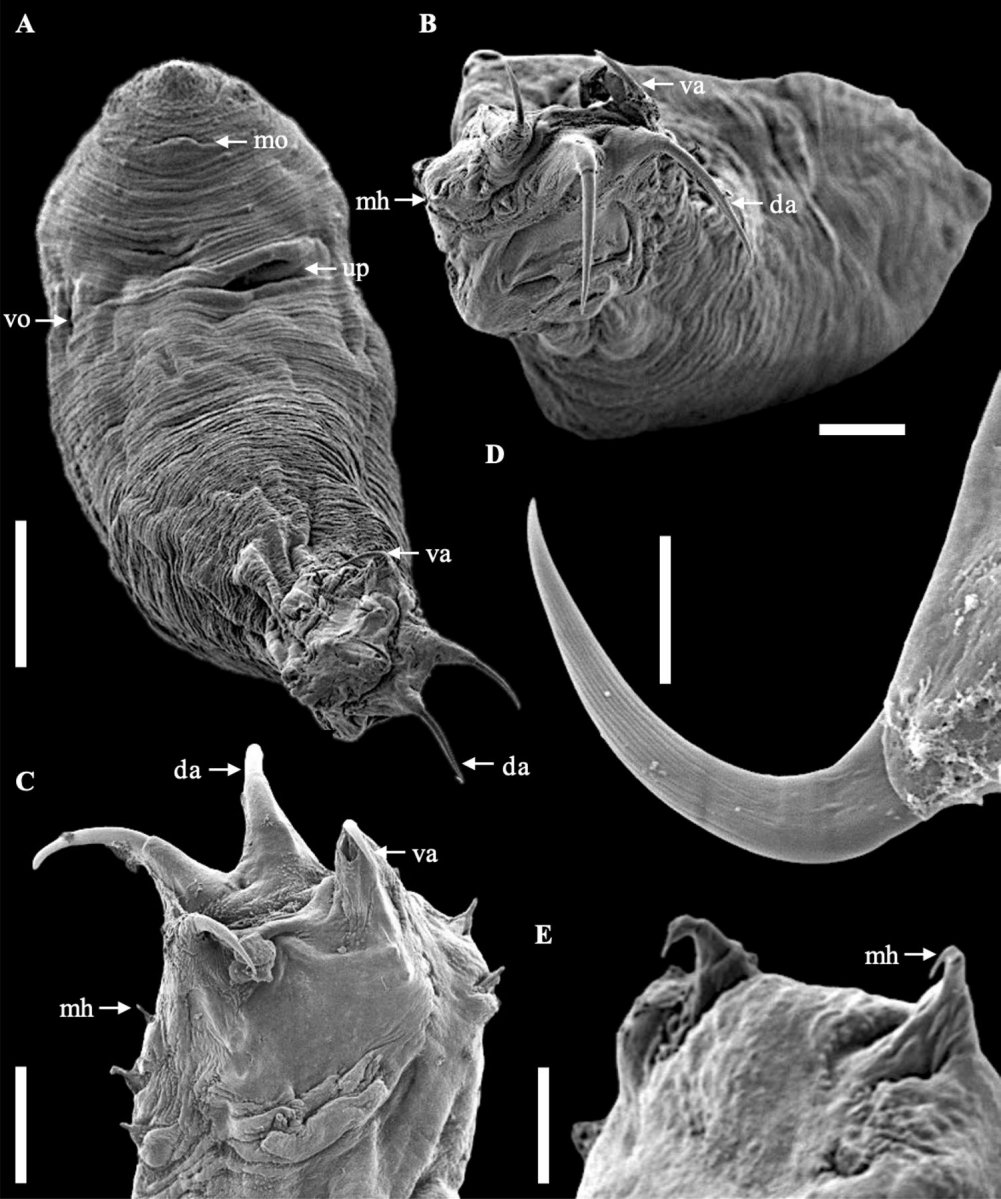
SEM micrographs of *Thaparocleidus vistulensis*. (**A**) Whole body—ventral view; (**B**) Whole body—posterior view; (**C**) Opisthaptor; (**D**) Dorsal anchor; (**E**) Marginal hooks. Abbreviations: da, dorsal anchor; mh, marginal hooks; mo, mouth opening; up, uterine pore; va, ventral anchor; vo, vaginal opening. Scale bars represent (**A**) 50 μm, (**B**–**C**) 20 μm, and (**D**-**E**) 5 μm.

**Figure 3:**
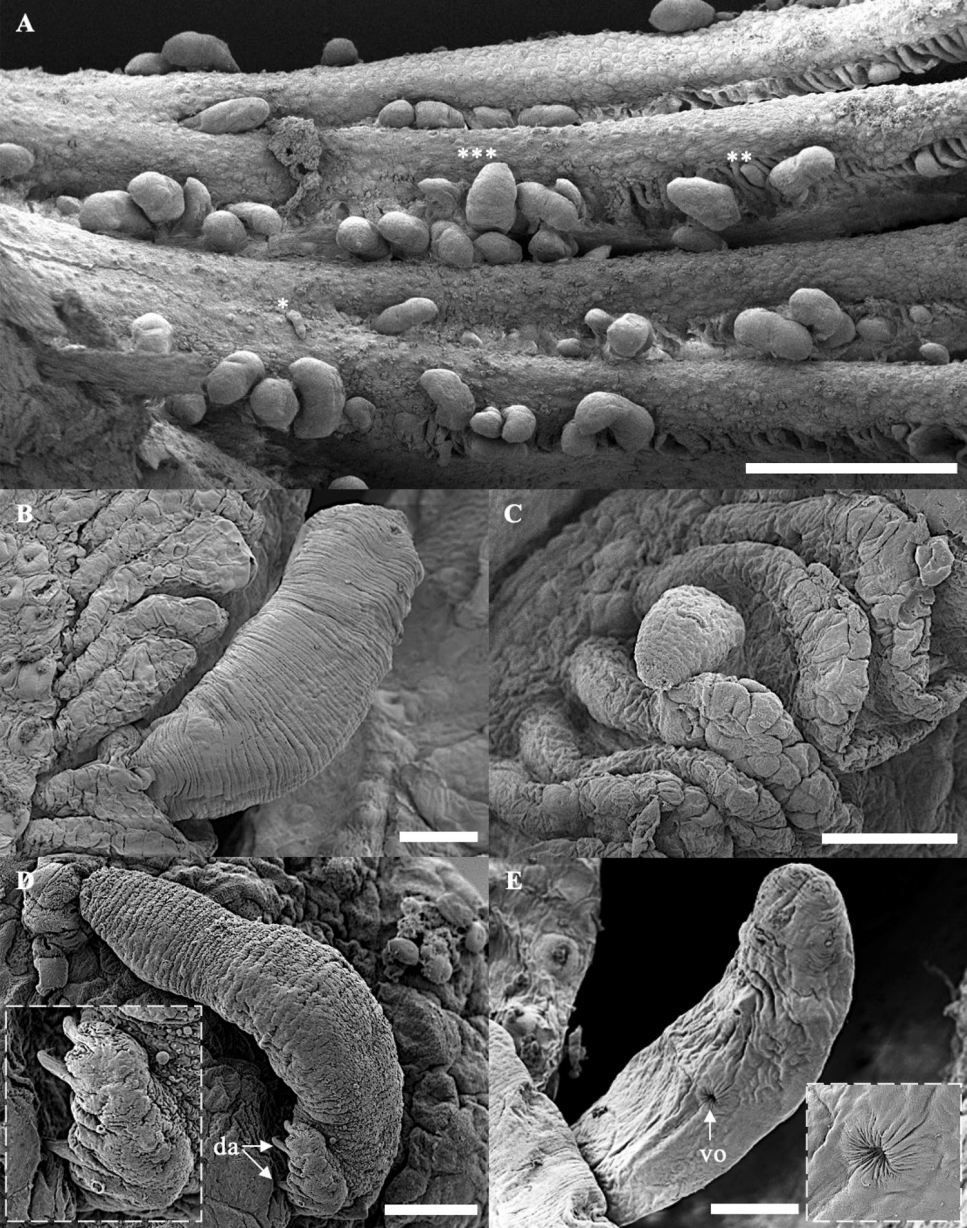
SEM micrographs of *Thaparocleidus vistulensis* are attached to gills lamellae. (**A**) Extensive hyperplasia of gill filament heavily infected by *T. vistulensis*; (**B**) Adult *T. vistulensis* with posterior part surrounded by gill tissue—lateral view; (**C**) Young *T. vistulensis* with posterior part in between gill lamellae—ventral view; (**D**) Adult *T. vistulensis* penetrating the gill filament—dorsal view; (**E**) Vaginal opening—ventral view. Abbreviations: da, dorsal anchor; vo, vaginal opening. Asterisks represent (*) Larvae, early stage of attachment, (**) Young, and (***) Adult *T. vistulensis*. Scale bars represent (A) 500 μm, (B, C, E) 50 μm, and (D) 20 μm.

**Figure 4:**
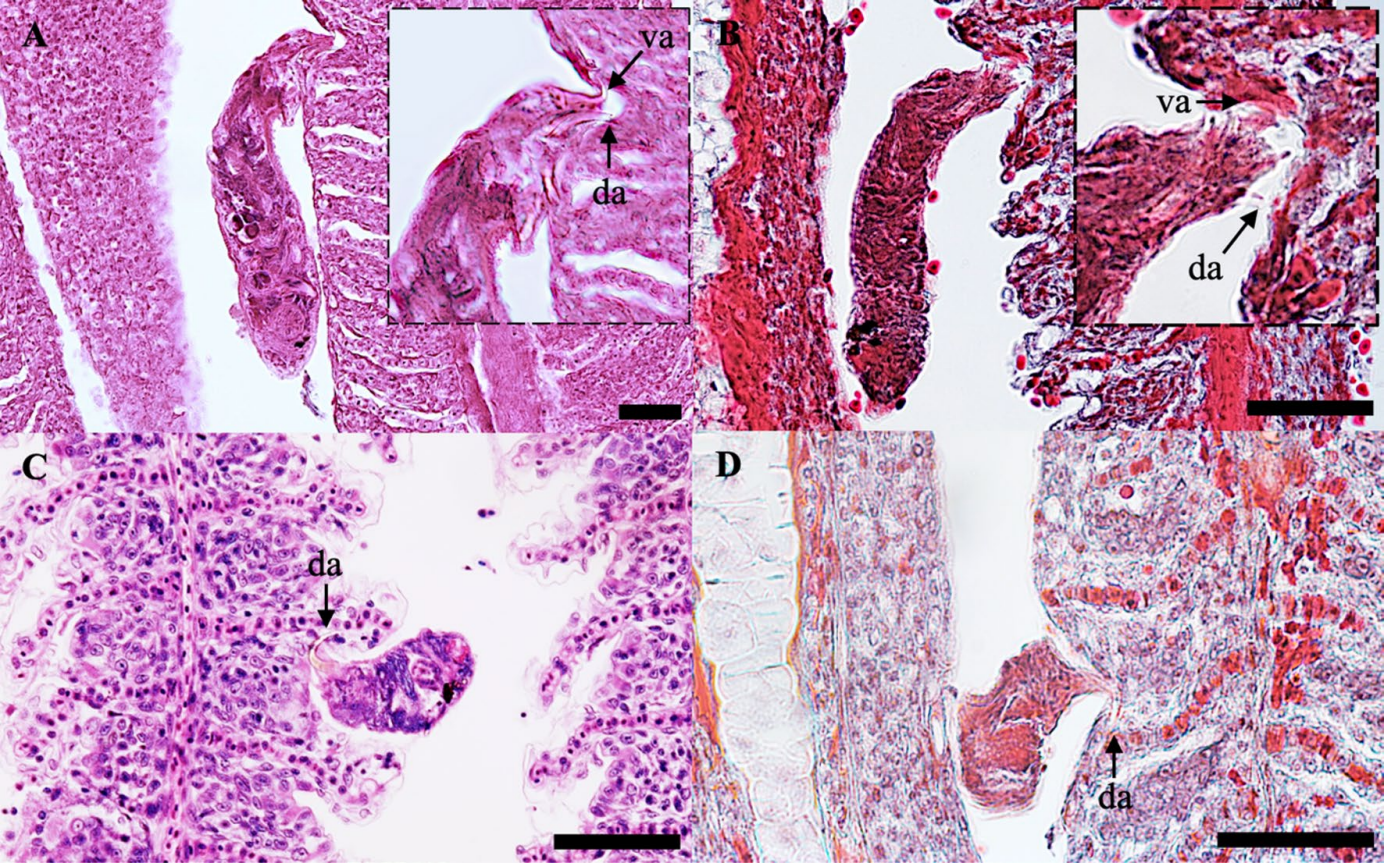
Histological sections of gills attached by *T. vistulensis*. (**A**-**B**) Adult *T. vistulensis* with posterior part—anchor inserted and pierced in gill lamellae in the opposite direction; (**C**-**D**) Young *T. vistulensis*. Staining method (**A**, **C**) stained with H & E; (**B**, **D**) stained with Masson–Goldner trichrome staining. Abbreviations: da, dorsal anchor; va, ventral anchor. Scale bars represent 50 μm.

The body is elongated and assumes a cylindrical form but tapers towards the posterior end and terminates with a slightly wider and non-segregated caudal disc (Fig. 1A and 2A–B). The anterior part of the body is extended and flattened, with two pairs of eyespots dorsally, and four pairs of head organs with cephalic glands (Fig. 1B-C). The eyes, aggregation of dispersed pigment spots are located along the vertices of a trapezoid. Adjacent to these pigment spots, positioned at a slightly oblique angle, transparent and intensely refractive corpuscles are discernible (Fig. 1C). The mouth opening is subterminal, and the pharynx, is roundish or short oval, located ventrally in the region behind the eyes (Fig. 1C). The widening haptoral region (Fig. 1G) has two pairs—dorsal and ventral—of strong anchors (Fig. 1H–I and 2C–D). The dorsal ones have well-developed inner roots and less prominent outer roots. The ventral anchors have a smaller size, having well-developed inner and outer roots. The dorsal anchors are connected with a straight dorsal bar, while the ventral ones are connected with a V-shaped ventral bar. A small sheet, called a cuneus, was observed joining the inner roots of the dorsal anchors. Both dorsal and ventral anchors are pointed in opposite directions (Fig. 1K–L, 2A–C and 4A–D). The haptor comprises 14 small marginal hooks (Fig. 1J and 2E). All the haptoral sclerites are provided with fine chitinous stirrups. The haptoral sclerites gripping the gill lamellae of their host can be seen in Fig. 3A–D. Vitellaria are densely dispersed throughout the trunk except in the region of reproductive organs. The testicle is located at the posterior part of the body and is connected with the vas deferens to the seminal vesicle. The seminal vesicle is single, blind-bottomed. The male copulatory organ starts with a flask-shaped bulb usually facing to the right of the ventral body position and connected to a long sclerotized penis tube (Fig. 1D). The flask-shaped bulb has 13.9 ± 0.6 (13.4–15.0) in length and 8.2 ± 1.1 (7.2–10.0) in width. The penis usually has 5–7 loops in the middle of its length before joining the penis accessory. The total length of the penis was 837.4 ± 95.9 (703.6–940.9). The accessory piece of the copulation organ is a sclerotized, open V-shaped structure, composed of a trough-like basal part which receives the penis. Its total length measures 97.0 ± 7.8 (92.5–110.9). In its middle where the accessory part turns to be V-shaped, it splits into two parts. One carries the penis, while the other has an elongated, slightly bent, and somewhat hook-like part that runs parallel before curving back towards the first. We often observed the penis running free on a short section after leaving the accessory piece.

The germarium (Fig. 1E) is anteroventral to the testis (Fig. 1F). Inside the germarium, large ovules with clear nuclei and nucleoli are present in the anterior part, while smaller cells can be seen in the posterior region. The size of the testis and germarium were not measured in the present study.

On the ventral side of the body, the vaginal opening is sinistral (Fig. 2A and 3E) and located above the germarium. The vaginal duct, with a measurement 358.1 ± 39.7 (323.2–409.1), consists of irregular convolutions, connecting to the uterine pore, often forming several loops, and ends at the opening of the seminal receptacle. The terminal part of the vagina forms a muscular chamber that encloses a small chitinous plaque. All features mentioned agree with the initial description of the species (Fig 4).

Oncomiracidia measure 167.3 ± 7.6 (157.5–177.6) in length and 72.5 ± 4.2 (65–78.4) in width. They have 14 marginal hooks with a length of 15.7 ± 0.7 (14.1–16.7), which are about the same size as those of matured worms. The sickle length was 5.1 ± 0.2 (4.7–5.6). Only an underdeveloped pair of ventral anchors was found in the attaching disc. Their length proved to be 21 ± 1.3 (19.2–22.7), with 2.6 ± 0.1 (2.5–2.8) inner root and 2.6 ± 0.2 (2.4–2.8) outer root.

### Remarks

Measurements of the sclerotized parts of organs in general corresponded to data from^8,25^, and^22^. Of them^8^, remarked that in young *T. vistulensis* specimens, the pair of ventral anchors appear and grow faster than the dorsal ones, we accept that anchors seen in oncomiracidia belonged to the future ventral anchors. *T. vistulensis* resembles *T. magnus* in the shape of a tube base with a flask-shaped, very long penis with several tubular loops in the middle of the penis, but differs in an accessory piece, where *T. magnus* has the appearance of a tuberous groove with a strongly swollen anterior end, forming three teeth of different shapes^15^. We completed the taxonomic characterization with the addition of SEM and histological figures, especially with the morphological description of the oncomiracidia of *T. vistulensis.*

### Molecular analysis and phylogenetic tree

In the present study, a 2694 bp long nucleotide sequence including 18S (partial), Internal Transcribed Spacer (ITS) 1, 5.8S, ITS2, and 28S (partial) ribosomal DNA (rDNA) of *Thaparocleidus vistulensis* was obtained. The sequence has been deposited in GenBank under the accession number OR916383. Due to the limited data available in the INSDC, only ITS1 sequences were involved in the phylogenetic analyses. The obtained sequence was then compared with previously deposited sequences of the genus *Thaparocleidus*. Our ITS1 sequence shares 97.96% identity with the *T. vistulensis* sequence identified in *Silurus glanis* from the Czechia (AJ490165) and exhibits a 92.18% similarity with *T. siluri* isolated from the same host species and geographical location (AJ490164)^27^.

**Figure 5:**
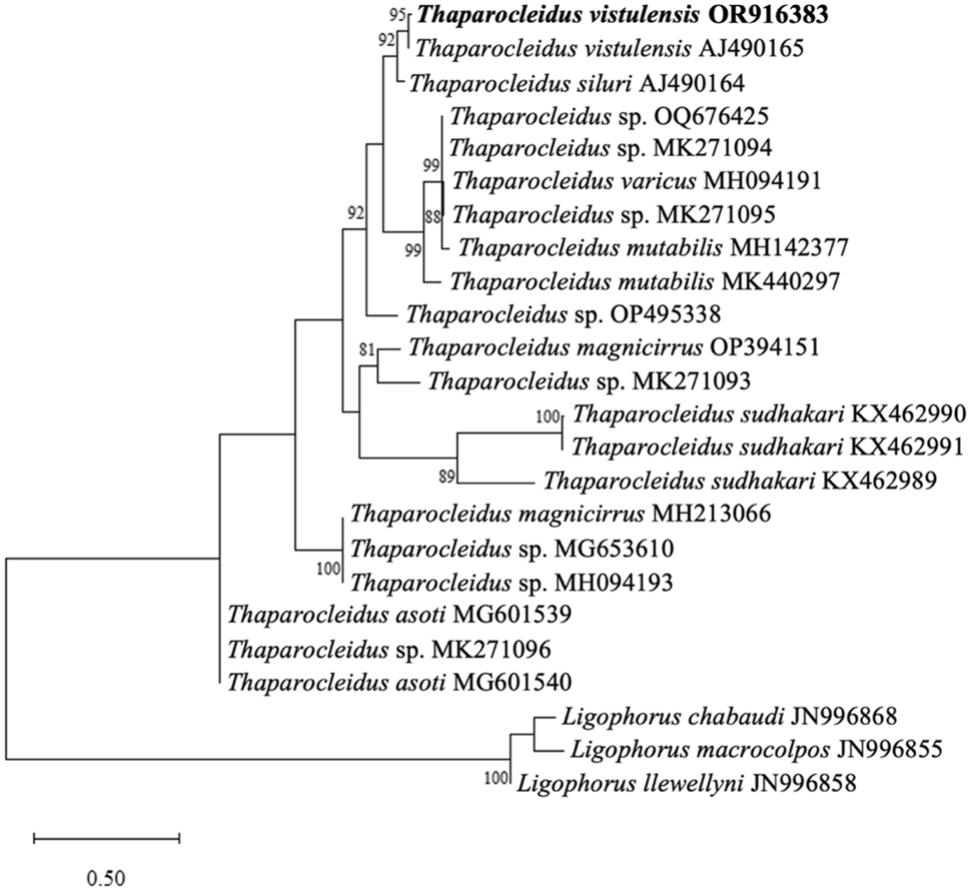
A phylogenetic constructed based on the ITS1 rDNA sequences demonstrating the positions of *Thaparocleidus vistulensis* with other *Thaparocleidus* species. The tree was generated by the ML method and rooted to *Ligophorus* spp. as an outgroup. Numbers at nodes indicate ≥ 80% bootstrap values (1000 replications). Species names are listed along the INSDC accession numbers. Species examined in this study are shown in bold. The scale bar indicates the number of expected substitutions per site.

The (maximum likelihood) ML tree, constructed based on the ITS1 rDNA, robustly supports a well-defined clade that includes the previously identified *T. vistulensis* sequence (Fig. 5). *Thaparocleidus vistulensis* forms a sister group with *T. siluri*, another parasitic species specific to *S. glanis*. Both *T. vistulensis* and *T. siluri* clustered with *T. varicus* and *T. mutabilis* in a distinct branch, and high bootstrap values strongly support this grouping. The sequence variabilities (uncorrected *p* distance) in the ITS1 within genus/species group with *Thaparocleidus vistulensis* were 52.3–98.3% (Supplementary Table 1). Molecular analyses provide compelling evidence that the monopisthocotylan species examined in this study can be confidently attributed to *T. vistulensis*.

## Discussion

The three related species (*Thaparocleidus siluri*, *T. vistulensis*, and *T. magnus*) were described from the European catfish by Zandt^28^, Siwak^8^, and Bychowsky & Nagibina^25^, respectively. These congeneric species were distinguished by several unique features^25^. The essential attributes of sclerotized structures, encompassing the haptoral parts and copulatory organs – copulatory piece and vagina, remain of paramount importance for species identification and taxonomy^29–34^. The morphological characteristics of the haptor are regarded as adequate for genus-level parasite identification, while the reproductive organ proves a more suitable clue for species-level discrimination, likely attributable to its higher rate of variability^31,35,36^. Therefore, it is vitally essential to describe these features and structures meticulously.

The body size of parasites in this study exhibits a broader range of length (507.1–1002.4) and total body width (120.6–196.2) compared to the literature. This variation can be attributed to the parasites being measured as early as day ten after infection, as indicated by^37^, who considered them to be in a mature stage. Furthermore, the measured monopisthocotylans were preserved in 80% ethanol for a period before assessment, potentially leading to a size reduction. Nevertheless, the dimensions of each sclerotized feature, specifically the attachment organ, were found to be approximately consistent with the previous study except smaller than those reported by^22^ (Table 1). This attachment organ suggests that the rigid components of the monopisthocotylan remain fixed and resistant to shrinking.

**Table 1:** Morphometric characteristics of *Thaparocleidus vistulensis* from the present study and relevant literature.

Morphometric characteristics	Siwak (1932)	Bychowsky and Nagibina (1957)	Paladini et al. (2008) (*n* = 20)	Present Study (*n* = 20)
Body Size *(*n* = 10)				
Total body length	(740.0–1140.0)	(400.0–750.0)	﻿1102.1 ± 167.6 (772.0–1325.6)	691.2 ± 163.0 (507.1–1002.4)
Oncomiracidia	–	–	–	167.3 ± 7.6 (157.5–177.6)
Total body width	(85.0–159.0)	(140.0–270.0)	﻿308.5 ± 48.8 (202.6–360.7)	155.2 ± 27.2 (120.6–196.2)
Oncomiracidia	–	–	–	72.5 ± 4.2 (65–78.4)
Dorsal anchor				
Total length	﻿(70.0–79.0)	(70.0–77.0)	﻿85.8 ± 2.4 (82.8–88.4)	66.2 ± 4.3 (57.6–73.9)
Shaft length	–	(58.0–63.0)	﻿71.7 ± 5.4 (66.2–81.6)	54.5 ± 3.5 (46.9–60.9)
Root length	﻿(18.0–22.0)	(16.0–19.0)	﻿17.7 ± 4.1 (10.5–23.3)	13.9 ± 1.5 (10.9–16.5)
Point length	–	(31.0–35.0)	﻿38.7 ± 3.2 (32.8–41.9)	31.4 ± 2.1 (27.3–35.0)
Aperture	﻿(59.0–68.0)	–	﻿52.8 ± 2.4 (49.1–56.5)	42.8 ± 3.6 (36.4–50.4)
Cuneus				
Total length	﻿(22.0–27.0)	(24.0–28.0)	﻿29.0 ± 2.1 (26.8–32.4)	23.4 ± 1.8 (19.7–26.5)
Largest width	–	(7.0–8.0)	﻿9.7 ± 1.5 (7.6–12.4)	7.8 ± 1.3 (5.8 –10.1)
Ventral Anchor				
Total length	﻿(27.0–30.0)	(25.0–28.0)	﻿30.3 ± 2.1 (27.3–32.7)	25.8 ± 1.2 (23.7–27.6)
Oncomiracidia *(*n* = 5)^a^	–	–	–	21 ± 1.3 (19.2–22.7)
Shaft length	–	(21.0–22.0)	﻿25.7 ± 1.3 (23.8–27.6)	21.1 ± 1.0 (19.3–22.6)
Inner root length	–	7.0	﻿8.7 ± 1.46 (7.0–11.2)	6.4 ± 0.6 (5.3–7.9)
Oncomiracidia *(*n* = 5)^b^	–	–	–	2.6 ± 0.1 (2.5–2.8)
Outer root length	–	–	–	5.3 ± 0.4 (4.5–6.0)
Oncomiracidia *(*n* = 5)^b^	–	–	–	2.6 ± 0.2 (2.4–2.8)
Point length	–	(14.0–16.0)	﻿16.2 ± 1.2 (14.3–17.8)	14.9 ± 1.0 (12.1–16.5)
Aperture	﻿(18.0–22.0)	–	﻿20.6 ± 1.6 (18.1–22.7)	18.1 ± 1.4 (16.0–21.2)
Oncomiracidia *(*n* = 5)^b^	–	–	–	14.8 ± 0.3 (14.6–15.3)
Dorsal bar				
Total length	﻿(32.0–37.0)	–	﻿38.4 ± 2.6 (35.1–41.9)	31.6 ± 2.4 (27.1–35.8)
Width in the middle	–	–	﻿9.32 ± 0.7 (8.1–10.0)	6.7 ± 1.2 (5.2–9.9)
Ventral Bar				
Length of one branch	﻿(23.0–25.0)	(23.0–25.0)	﻿25.7 ± 1.5 (23.3–27.5)	21.9 ± 1.4 (19.1–24.4)
Largest width	–	3.0	﻿5.3 ± 0.8 (4.3–6.8)	3.1 ± 0.5 (2.4–4.5)
Marginal Hook *(*n* = 40)				
Total length	﻿16.3	16.0	﻿17.5 ± 0.5 (16.8–17.9)	15.8 ± 0.6 (14.9–16.9)
Oncomiracidia	–	–	–	15.7 ± 0.7 (14.1–16.7)
Sickle length	﻿4.3	–	﻿6.3 ± 0.4 (5.7–6.8)	5.3 ± 0.3 (4.8–5.9)
Oncomiracidia	–	–	–	5.1 ± 0.2 (4.7–5.6)
Male copulatory organ *(*n* = 5)				
Penis	–	640.0	–	837.4 ± 95.9 (703.6–940.9)
No. of loop	–	≥ 4	–	5–7
Accessory piece	–	(68.0–71.0)	–	97.0 ± 7.8 (92.5–110.9)
Farthest point	–	–	–	131.9 ± 20.4 (103.4–156.0)
Bulbous base length	–	(14.0–16.0)	–	13.9 ± 0.6 (13.4–15.0)
Bulbous base width	–	8.0	–	8.2 ± 1.1 (7.2–10.0)
Female copulatory organ *(*n* = 5)				
Vaginal duct	–	≈ 200	–	358.1 ± 39.7 (323.2–409.1)

The number of studied parasites and mean values of morphometric characters were not specified in^8^, and^25^. Mean ± Standard deviation, with range in parentheses. Measurements expressed in micrometers (μm).

*Referred to the number of specimens examined in the present study.

^a^Morphological part was measured following the anchor curve.

**Figure 6:**
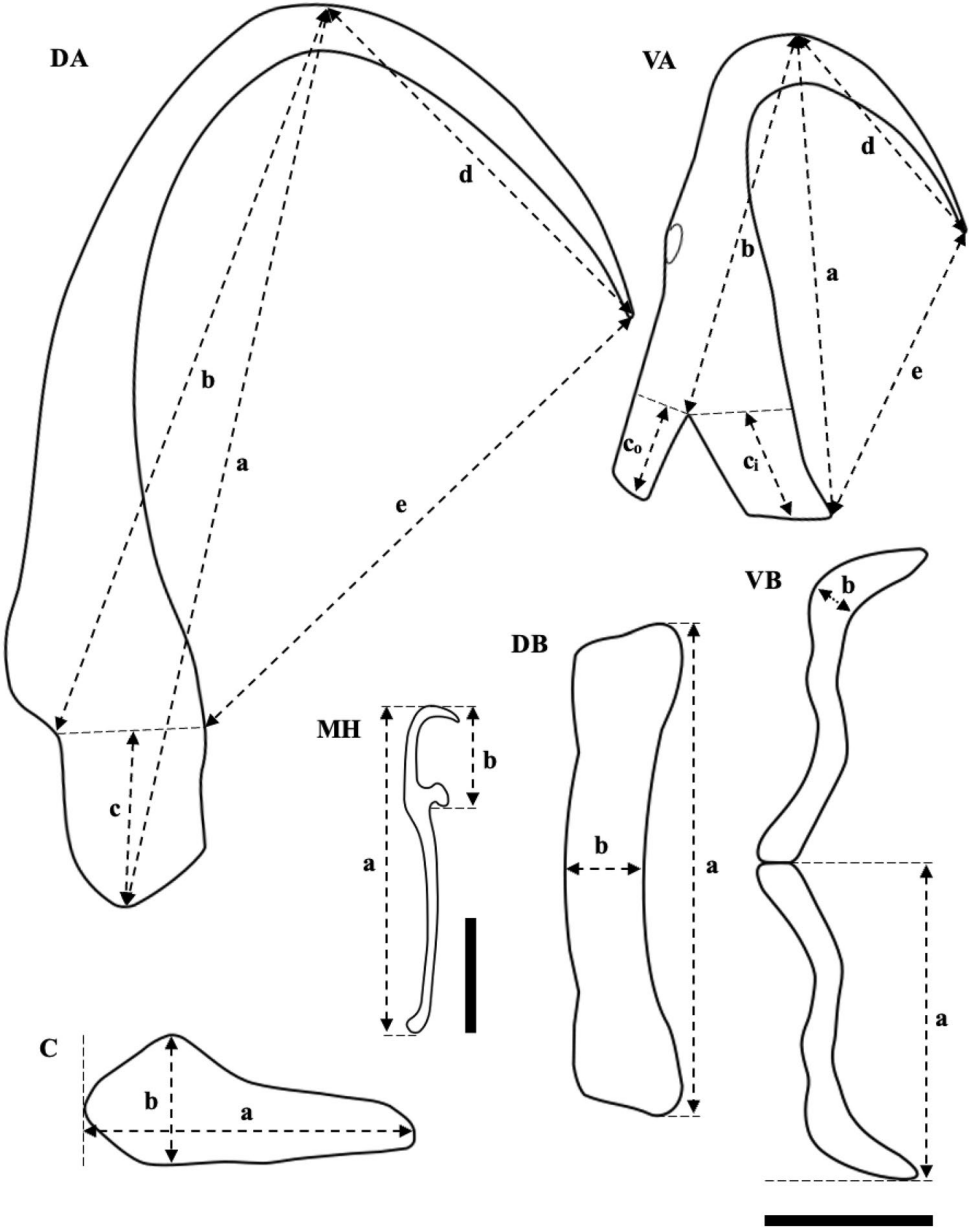
Metric parameters of the *Thaparocleidus vistulensis* attachment apparatus used in this study. Abbreviations: C, cuneus (a, total length; b, largest width); DA, dorsal anchor (a, total length; b, shaft length; c, root length; d, point length; e, aperture); DB, dorsal bar (a, total length; b, width in the middle); MH, marginal hooks (a, total length; b, sickle length); VA, ventral anchor (a, total length; b, shaft length; c_i_, inner root length; c_o_, outer root length; d, point length; e, aperture); VB, ventral bar (a, length of one branch; b, largest width). All parts of anchors refer to a scale bar of 10 μm, except for the marginal hooks, with a scale bar of 5 μm. The terminology and methodology of measurements are according to^29,50^, and^22^.

^b^Morphological part was measured following ventral anchor parameters in Fig. 6.

The male copulatory organ of *T. vistulensis* is a relatively large structure. Hitherto, only a few studies included the dimension size of the copulatory organ studied by light microscopy in whole-mounted specimens^25,38^. Furthermore, although some of these authors have provided detailed descriptions of an excellent drawing, the exact measurement points are not mentioned, making it sometimes difficult to interpret their descriptions. The present report of the male copulatory organ is based on light microscopy with a detailed point of measurement on the organ’s features, allowing a detailed analysis of the shape.

Assessing from the published drawings, at least eight *Thaparocleidus* species possess a male copulatory organ (i.e., a long thread-like sclerotized penis with coils) similar to that of *T. vistulensis*. This characteristic is shared with several other species, as outlined in Table 2. The overall structure of the male copulatory organ bears a resemblance to *T. magnus* and *T. siluri*, characterized by a sclerotized penis tube structure with a flask-shaped bulb, coils in the middle of the length of the penis, and an accompanying accessory piece. However, *T. vistulensis* exhibits a closer affinity to *T. magnus*, but is easily distinguishable based on the male copulatory organ (i.e., distant points of the copulatory organ) other than the size of body and attachment structure^25^.

**Table 2:** List of freshwater monopisthocotylan parasites with long penis and coils based on published drawings.

Species	Total length	Total coils	References
Genus *Thaparocleidus* Jain 1952			
* T. armillatus* Verma, Chaudhary and Singh, 2017	82–89	1.5	^51^
* T. devraji* Gusev, 1976	93–133	1.5	^52^
* T. magnus* Bychowsky and Nagibina, 1957	1400–1600	*	^25^
* T. malabaricus* Gusev, 1976	*	3.5	^53^
* T. seenghali* Jain, 1961	*	2–3	^53^
* T. siluri* Zandt, 1924	390–420	2–3	^25^
* T. susanae* Rajvanshi & Agrawal, 2013	193–198	3	^54^
* T. wallagonius* Jain, 1952	*	3–4	^51^
Genus *Demidospermus* Suriano, 1983			
* D. spirophallus* Franceschini, Zago, Müller, Francisco, Takemoto & da Silva, 2017	193–230	2.5	^26^
* D. prolixus* Franceschini, Zago, Müller, Francisco, Takemoto & da Silva, 2017	210–234	1.5	^26^
* D. anus* Suriano, 1983	143–156	1–1.5	^26^
Genus *Mastacembelocleidus* Kritsky, Pandey, Agrawal & Abdullah, 2004			
* M. bam* Tripathi, 1959	*	2	^55^
* M. heteranchorus* Kulkarni, 1969	*	2	^55^
Genus *Dactylogyrus* Diesing, 1850			
* D. nasutai* Narba, Matey, Agarwal & Tripathi, 2022	*	27	^56^
* D. pulcher* Bychowsky, 1957	250	6–7	^39^
* D. simplicimalleata* Bychowsky, 1931	340	*	^39^
* D. wuhuensis* Lee, 1960	155–185	2.5–3	^39^
* D. falciformis* Akhmerov, 1952	190–220	3	^39^
* D. procypris* Ma, Li & Wang, 1981	360	*	^57^
* D. longivagina* Zhang & Pan, 1988	410–610	6–8	^57^
* D. pseudoflagillicirrus* Long, 1964	300	*	^57^
* D. luciosomis* Zhang & Guo, 1981	80–140	*	^57^
* D. sphyrna* Linstow, 1878	90–98	*	^57^
* D. onychocirrus* Long, 1981	88—110	*	^57^
* D. lingualis* Long, 1981	114–125	3	^57^
* D. rhychoideus* Long, 1981	122	2	^57^
* D. spirovagina* Long, 1981	71	2	^57^
* D. longquanensis* Wu & Wang, 1983	199–282	1–2	^57^
* D. quadricurvitubus* Zhang & Guo, 1982	165–235	4–7	^57^
* D. austrosinensis* Zhang & Li, 1991	190–207	2	^57^
* D. strombus* Tao & Long, 1981	340–660	4–11	^57^
* D. daojiensis* Luo & Long, 1982	376	*	^57^
* D. pectinate* Zhao & Ma, 1991	174–177	2	^57^
* D. ehrenbergii* Yao & Wang, 1997	149–210	2	^57^
* D. garrae* Ma & Long, 2000	167–185	7	^57^
* D. lianshanensis* Ma & Long, 2000	120–207	*	^57^
* D. helicoides* Yao & Wang, 1997	376–1455	4–10	^57^
Genus *Pseudacolpenteron* Bychowsky & Gusev, 1955			
* P. ignotus* Gussev, 1955	190	2	^39^
Genus *Ancyrocephalus* Creplin, 1839			
* A. subaequalis* Akhmerov, 1952	130–170	*	^39^
* A. pavlovskyi* Gussev, 1955	140–160	*	^39^
* A. brevifilis* Yao & Wang, 1997	248–348	*	^57^
Genus *Dogielius* Bychowsky, 1936			
* D. strombicinms* Ma & Long, 2000	357	7	^57^
Genus *Pseudancylodiscoides* Yamaguti, 1963			
* P. panduriformis* Zhang & Ma, 1997	116–149	1.5	^57^

*Data not provided.

In certain instances, taxonomic identification presents challenges at both generic and specific taxonomic levels. As a result, molecular identification emerges as a valuable tool for clarifying taxonomic issues, especially when morphological distinctions among monopisthocotylan genera or species groups are ambiguous^31,35,36^. The phylogenetic analysis clustered the *T. vistulensis* sequence obtained in this study (Fig. 5) together with other *Thaparocleidus* spp. that have shown the highest nucleotide similarities via a BLAST search.

In the present study, a 2694 bp-long fragment, including partially 18S, ITS, 5.8S, and partially 28S rDNA of the monopisthocotylan (accession number OR916383), was successfully sequenced. A lack of available *Thaparocleidus* species sequences covering all these gene markers in the GenBank prevented the alignment of the whole sequence generated. Therefore, a part of the obtained sequence was removed before analysis, and only ITS1 was used for the comparison. The ITS1 sequences of two *T. vistulensis* isolates from the current and previous studies (AJ490165) are found to be closely related to each other. In the phylogenetic tree, both isolates branched at a single nodal point well supported by high bootstrap, proving they belong to the same species. *T. siluri* was found to be a sister species to *T. vistulensis*. This clade comprises *Thaparocleidus* species that have been reported to infect *S. glanis*^25,39^.

Based on the morphological characterization and molecular analysis of the ITS1, we can conclude that the studied monopisthocotylan species is *T. vistulensis,* which was previously widely reported to infect *S. glanis*. This study provides a redescription for *T. vistulensis*, particularly for the characterization of the male copulatory organ, combined with the molecular data for species identification. To better understand this monopisthocotylan parasite, further studies on multiple parasite specimens and genetic markers are needed. In addition, the pathogen-specific effect of *T. vistulensis* on the host gills would be worth discussing in a forthcoming study, as to our knowledge, no information on this is currently available in the literature, except for Molnár^40^.

## Methods

### Ethical statement

The experimental protocols together with fish handling and sampling were approved by the Institutional Animal Care and Use Committee of the Veterinary Medical Research Institute, Budapest, Hungary. All research involving experiments on fish (European catfish) was reviewed and approved by the Hungarian National Scientific Ethical Committee on Animal Experimentation under reference number: PE/EA/00081-4/2023. The authors complied with the ARRIVE guidelines (https://arriveguidelines.org).

### Collection of fish and parasites

Naturally infected European catfish with *Thaparocleidus vistulensis*, obtained from a commercial fish farm in Hungary, were transported to the Veterinary Medical Research Institute in Budapest, Hungary (HUN-REN VMRI), maintained at 23 ± 1 °C in a flow-through tank system, and subjected to a parasitological investigation. Upon arrival at the institute, gill biopsies were procured from the first gill arch using dissection scissors^41^ to confirm the presence of parasites. The parasite population was then maintained by co-habitation according to the method of^42^, and the adult and larval (oncomiracidia) parasites were collected following^37^. All life stages of monopisthocotylan were fixed in 80% ethanol and 5% formalin. Some parasites were freshly recovered and studied alive, but additional samples, left and right sides of excised gill arches, were preserved in 80% ethanol or 5% formalin, respectively, until further use.

### Morphological analysis

Some parasites were softened and cleared in a mild enzymatic digestion proteinase K, modifying the method described by^43^, before mounted individually in 1–2 drops of glycerine-ammonium-picrate on a slide (depending on the size of specimens)^44^. The preparation was then covered with a coverslip. Some specimens were stained using hematoxylin (Harris’ modified solution, Sigma-Aldrich HHS32), mounted on a glass slide using AQUATEX® (cat. no. HC568794, Merck), and covered by a coverslip.

**Figure 7:**
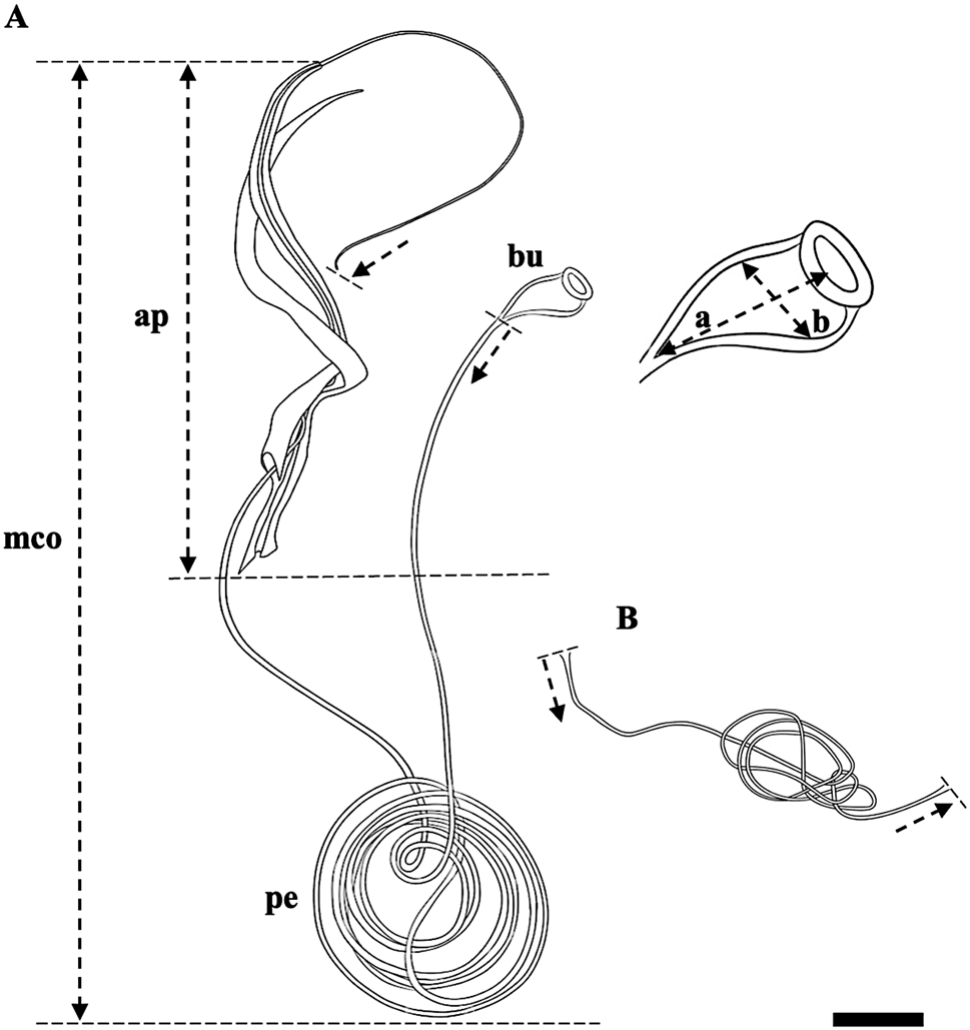
Measurements of the male copulatory organ. (A) Male copulatory organ; (B) Vaginal duct. Abbreviations: ap, total length of accessory piece; bu, bulbous base (a, total length; b, largest width); mco = distal end points of the male copulatory organ; pe, total length of penis (|→ = starting point, →| = ending point). Scale bar represents 20 μm.

Photomicrographs were performed using a digital camera (Leica MC170 HD) with LAS V4.12 software equipped with a light microscope (Leica DM5000B Microscope W/ CTR5000 Controller). Subsequently, line drawings of the monopisthocotylan’s important part (e.g., the sclerotized structure of the haptor and the male copulatory complex) were made based on the photos. The measurements were made and analyzed based on the captured images using the scientific image analysis tool—ImageJ 1.53t software (RRID: SCR_003070). The morphological parameters of monopisthocotylan, including the sclerotized structure and male copulatory organ, were measured as proposed by^45^ and^22^ (Figs. 6 and 7). These measurements (Table 1) were presented as mean with standard deviation followed by the range in parenthesis, provided in micrometers (μm). Measurements were conducted of adult parasites: total body width and length (*n* = 10), each of attachment structures (*n* = 20), marginal hooks (*n* = 40), and copulatory organs (*n* = 5). Structures of oncomiracidia (*n* = 5) (except for marginal hooks) were measured as well. Data obtained from this study were compared with the previous descriptions by^8,25^, and^22^.

### Scanning electron microscope (SEM)

The parasites that were previously preserved in 5% formalin were transferred in Karnovsky’s fixative (4% paraformaldehyde and 2% glutaraldehyde in 0.1 M sodium cacodylate buffer) for 15-min, then rinsed in 0.1 M sodium cacodylate buffer two times for 20-min, before immersed in demineralized water two times for 15-min. The samples were then dehydrated through the ascending concentrations of ethanol series (20, 30, 50, 70, 90, 90, 100, and 100%) for 15-min per treatment, except 100% (30-min per treatment). Samples were subsequently dried by passing into 100% hexamethyldisilazane (HMDS) (cat. no. 52620, Fluka) for 30-min and set aside in a fume hood overnight. The dehydrated samples were attached to a strip of carbon conductive double-sided tape that was fixed to an SEM aluminum stub. Then, the samples were sputter coated with gold in Leica EM ACE200 Vacuum Coater (Leica, Wetzlar, Germany) with a thickness of 5–10 nm and examined in an FEI Quanta 200 SEM (FEI Company, Hillsboro, Oregon, United States) operating at 3–8 kV acceleration voltage, using xT Microscope Control software.

### Histology

For histology, the gills arch infected with monopisthocotylans that were previously fixed in 5% formalin solution were processed by standard histology techniques, dehydrated in a series of ethanol (70, 96, 100%) and xylol baths, embedded in paraffin wax for cross sections at a 5 µm thickness using a microtome (Leica RM2135, Germany). Sections were deparaffinized before being stained with hematoxylin–eosin (H&E) and Masson–Goldner trichrome staining. Histological sample sections were observed under a light microscope.

## Molecular identification and phylogenetic analyses

Species identification of monopisthocotylans was confirmed by molecular methods, PCR and sequencing. Adult monopisthocotylans that were preserved in 80% ethanol were used. The DNA of the specimen was extracted using a QIAamp® DNA Mini Kit (250) (cat. no. 51306, Qiagen, Denmark) according to the manufacturer’s protocol with a final elution volume of 50 µl. The extracted DNA (2 µl) was subsequently quantified using a NanoDrop (ND-1000) spectrophotometer (Thermo Fisher Scientific, Wilmington, DE, USA). The ITS rDNA fragment encompassing ITS1, 5.8S, ITS2 and flanked with 18S and 28S rDNA genes were amplified with primers PDG_18S_F5 (5’- CGA TAA CGA ACG AGA CTC—3’) (in house primer) and NLR1270 (5’—TTC ATC CCG CAT CGC CAG TTC—3’)^46^. The PCR amplification was performed on a T100 Thermal Cycler (Bio-Rad, Hercules, CA, USA) with a total volume of 60 µl reaction mixture containing 6 μl sample DNA, 6 μl for each primer (10 mM), 6 µl 10 × NH_4_ buffer, 1.8 μl MgCl_2_ (50 mM), 0.6 μl DNA polymerase 5 U/µl (cat. no BIO-21060, Nordic BioSite, Denmark), 6 μl dNTP’s (10 mM) (cat. no. 4303442 Thermo Fisher Scientific, Denmark), and UltraPure™ DNase/RNase-Free Distilled Water (cat. no. 10977049, Thermo Fisher Scientific, Denmark)^47^. The PCR reaction conditions were 5-min at 94 °C for initial denaturation, followed by 45 cycles of denaturation at 94 °C for 30-s, annealing at 54 °C for 30-s, extension at 72 °C for 2.5-min, with a final extension step at 72 °C for 7-min, and an indefinite hold at 4 °C. The PCR product was expected to be 2694 bp. The PCR product was separated by gel electrophoresis in 1.5% agarose (cat. no. 10264544, Thermo Fisher Scientific, Denmark) Tris–acetate-EDTA (TAE) gel containing ethidium bromide stain alongside 5 μl of a 50 bp DNA Hyperladder™ (cat. no BIO-33040, Nordic BioSite, Denmark), and the amplified DNA fragment was visualized under Azure 200 Gel Imaging Workstation (Azure Biosystems, Dublin, California, USA). The PCR product was purified using Illustra™ GFX™ PCR and Gel band purification kit (VWR International, Denmark), and sequencing was performed at Macrogen Europe BV (Amsterdam, Netherlands) using the same PCR primers. Sequences obtained were analyzed using CLC Main Workbench v20.0.4 software (Qiagen, Denmark). Afterwards, the sequence was confirmed by BLAST analysis at the National Center for Biotechnology Information (NCBI) platform. The sequence was then submitted to GenBank. For the phylogenetic analysis, only the ITS1 sequence region was selected due to limited data availability for other parts of the rDNA of *Thaparocleidus* spp. in the INSDC. For molecular comparison, 20 ITS1 sequences of related species were chosen from the databases. Sequences were aligned using ClustalW algorithm in MEGA 11^48^. The phylogenetic tree was performed by the ML method using the general time-reversible (GTR + G + I) substitution model according to the Akaike information criterion (AIC) in MEGA 11^49^. Bootstrap analysis with 1000 replicates was applied to estimate nodal support. The analysis involved 24 nucleotide sequences with a total of 293 positions in the final data sets for the ITS1 gene marker. Sequences of *Ligophorus llewellyni* (JN996858), *Ligophorus chabaudi* (JN996868) *Ligophorus macrocolpos* (JN996855) were used to root the phylogenies. Level of sequence variation based on uncorrected pairwise distance (*p* distance) was calculated using MEGA 11^48^.

### Supplementary Information

Supplementary Information.

## Data Availability

Raw sequencing data that support the findings of this study have been deposited to the database of NCBI with accession number OR916383, while the datasets generated and analysed during the current study are available from the corresponding author on reasonable request.
