# Supplementary material for: Molecular and SEM studies on Thaparocleidus vistulensis (Siwak, 1932) (Monopisthocotyla, Ancylodiscoididae)
Source: Sci Rep. 2024 May 4;14:10292. doi: 10.1038/s41598-024-61032-3 (PMC11069529; doi:10.1038/s41598-024-61032-3)
Supplement: Supplementary file 1 — Supplementary Information. [file 41598_2024_61032_MOESM1_ESM.docx]

Table 1 Distances (in %) between taxa (p-distances)

|  | Accession No. ID, Species and Host | 1 | 2 | 3 | 4 | 5 | 6 | 7 | 8 | 9 | 10 | 11 | 12 | 13 | 14 | 15 | 16 | 17 | 18 | 19 | 20 | 21 | 22 | 23 |
| --- | --- | --- | --- | --- | --- | --- | --- | --- | --- | --- | --- | --- | --- | --- | --- | --- | --- | --- | --- | --- | --- | --- | --- | --- |
| 1 | OR916383 *Thaparocleidus vistulensis* ex *Silurus glanis* |  |  |  |  |  |  |  |  |  |  |  |  |  |  |  |  |  |  |  |  |  |  |  |
| 2 | AJ490165 *Thaparocleidus vistulensis* ex *Silurus glanis* | 98.3 |  |  |  |  |  |  |  |  |  |  |  |  |  |  |  |  |  |  |  |  |  |  |
| 3 | AJ490164 *Thaparocleidus siluri* ex *Silurus glanis* | 92.8 | 93.9 |  |  |  |  |  |  |  |  |  |  |  |  |  |  |  |  |  |  |  |  |  |
| 4 | OQ676425 *Thaparocleidus* sp. | 79.9 | 79.6 | 80.2 |  |  |  |  |  |  |  |  |  |  |  |  |  |  |  |  |  |  |  |  |
| 5 | MH094191 *Thaparocleidus varicus* | 79.9 | 79.6 | 69.9 | 99.0 |  |  |  |  |  |  |  |  |  |  |  |  |  |  |  |  |  |  |  |
| 6 | MK271094 *Thaparocleidus* sp. ex *Silurus asotus* | 79.9 | 79.6 | 80.2 | 100.0 | 99.0 |  |  |  |  |  |  |  |  |  |  |  |  |  |  |  |  |  |  |
| 7 | MK271095 *Thaparocleidus* sp. ex *Silurus asotus* | 79.9 | 79.6 | 69.9 | 99.0 | 99.3 | 99.0 |  |  |  |  |  |  |  |  |  |  |  |  |  |  |  |  |  |
| 8 | MH142377 *Thaparocleidus mutabilis* | 78.5 | 78.2 | 78.8 | 97.4 | 96.4 | 97.4 | 96.4 |  |  |  |  |  |  |  |  |  |  |  |  |  |  |  |  |
| 9 | MK440297 *Thaparocleidus mutabilis* ex *Silurus asotus* | 79.4 | 69.7 | 69.7 | 89.5 | 88.5 | 89.5 | 88.5 | 87.6 |  |  |  |  |  |  |  |  |  |  |  |  |  |  |  |
| 10 | OP394151 *Thaparocleidus magnicirrus* ex *Silurus asotus* | 74.8 | 64.6 | 73.8 | 70.0 | 69.3 | 70.0 | 69.3 | 69.3 | 73.3 |  |  |  |  |  |  |  |  |  |  |  |  |  |  |
| 11 | MK271093 *Thaparocleidus* sp. ex *Silurus asotus* | 72.0 | 71.4 | 71.7 | 67.8 | 67.1 | 67.8 | 67.1 | 67.1 | 70.1 | 81.9 |  |  |  |  |  |  |  |  |  |  |  |  |  |
| 12 | MG601539 *Thaparocleidus asoti* | 69.2 | 67.9 | 68.5 | 64.8 | 64.8 | 64.8 | 64.8 | 64.6 | 64.1 | 65.6 | 65.9 |  |  |  |  |  |  |  |  |  |  |  |  |
| 13 | MG601540 *Thaparocleidus asoti* | 69.2 | 67.9 | 68.5 | 64.8 | 64.8 | 64.8 | 64.8 | 64.6 | 64.1 | 65.6 | 65.9 | 100.0 |  |  |  |  |  |  |  |  |  |  |  |
| 14 | MK271096 *Thaparocleidus* sp. ex *Silurus asotus* | 69.2 | 67.9 | 68.5 | 64.8 | 64.8 | 64.8 | 64.8 | 64.6 | 64.1 | 65.6 | 65.9 | 100.0 | 100.0 |  |  |  |  |  |  |  |  |  |  |
| 15 | MH213066 *Thaparocleidus magnicirrus* | 70.1 | 69.8 | 69.3 | 69.1 | 69.1 | 69.1 | 68.4 | 68.5 | 68.1 | 71.2 | 70.1 | 70.9 | 70.9 | 70.9 |  |  |  |  |  |  |  |  |  |
| 16 | MH094193 *Thaparocleidus* sp. | 69.9 | 69.5 | 69.4 | 68.9 | 68.9 | 68.9 | 68.2 | 68.3 | 67.9 | 71.0 | 70.3 | 71.0 | 71.0 | 71.0 | 100.0 |  |  |  |  |  |  |  |  |
| 17 | MG653610 *Thaparocleidus* sp. | 70.1 | 69.8 | 69.3 | 69.1 | 69.1 | 69.1 | 68.4 | 68.5 | 68.1 | 71.2 | 70.1 | 70.9 | 70.9 | 70.9 | 100.0 | 100.0 |  |  |  |  |  |  |  |
| 18 | OP495338 *Thaparocleidus* sp. ex *Silurus asotus* | 82.1 | 82.1 | 83.4 | 75.8 | 75.1 | 75.8 | 74.7 | 75.4 | 77.3 | 75.8 | 72.3 | 65.6 | 65.6 | 65.6 | 69.3 | 69.0 | 69.3 |  |  |  |  |  |  |
| 19 | KX462990 *Thaparocleidus sudhakari* ex *Wallago attu* | 52.5 | 52.1 | 52.7 | 52.3 | 51.5 | 52.3 | 52.3 | 52.3 | 54.2 | 59.8 | 53.9 | 52.1 | 52.1 | 52.1 | 54.0 | 53.8 | 54.0 | 55.2 |  |  |  |  |  |
| 20 | KX462991 *Thaparocleidus sudhakari* ex *Wallago attu* | 52.3 | 51.7 | 52.3 | 51.9 | 51.1 | 51.9 | 51.9 | 51.9 | 54.1 | 59.3 | 53.5 | 52.5 | 52.5 | 52.5 | 53.8 | 53.6 | 53.8 | 55.0 | 98.5 |  |  |  |  |
| 21 | KX462989 *Thaparocleidus sudhakari* ex *Wallago attu* | 56.7 | 56.3 | 57.6 | 57.9 | 57.9 | 57.9 | 57.1 | 59.0 | 57.5 | 60.8 | 58.5 | 56.7 | 56.7 | 56.7 | 59.7 | 59.9 | 59.7 | 60.7 | 63.1 | 63.1 |  |  |  |
| 22 | JN996868 *Ligophorus chabaudi* ex *Mugil cephalus* | 38.4 | 37.9 | 39.8 | 36.6 | 37.1 | 36.6 | 36.6 | 37.1 | 37.2 | 37.4 | 37.2 | 41.2 | 41.2 | 41.2 | 39.3 | 39.3 | 39.3 | 37.2 | 34.2 | 34.2 | 36.9 |  |  |
| 23 | JN996858 *Ligophorus llewellyni* ex *Liza haematocheilus* | 41.1 | 40.6 | 44.2 | 37.5 | 37.9 | 37.5 | 37.5 | 37.9 | 37.6 | 37.0 | 37.6 | 40.9 | 40.9 | 40.9 | 39.8 | 39.8 | 39.8 | 39.3 | 33.2 | 33.2 | 39.3 | 87.5 |  |
| 24 | JN996855 *Ligophorus macrocolpos* ex *Liza saliens* | 37.6 | 37.2 | 38.7 | 34.1 | 35.0 | 34.1 | 35.0 | 34.5 | 34.2 | 34.5 | 34.2 | 38.5 | 38.5 | 38.5 | 36.1 | 36.1 | 36.1 | 36.1 | 32.9 | 32.9 | 36.1 | 86.0 | 86.1 |
